# Complete Genome Sequence of Dickeya chrysanthemi Bacteriophage DchS19

**DOI:** 10.1128/mra.00800-22

**Published:** 2022-09-14

**Authors:** Prasanna Mutusamy, Sasireigga Jaya Jothi, Yukgehnaish Kumarasan, Stella Loke, Su Yin Lee, Heera Rajandas, Sivachandran Parimannan

**Affiliations:** a Centre of Excellence for Omics-Driven Computational Biodiscovery, AIMST University, Bedong, Kedah, Malaysia; b Department of Biotechnology, Faculty of Applied Sciences, AIMST University, Semeling, Kedah, Malaysia; c Section for Evolutionary Genomics, Globe Institute, Faculty of Health and Medical Sciences, University of Copenhagen, Copenhagen, Denmark; d Deakin Genomics Centre, School of Life and Environmental Sciences, Faculty of Science, Engineering, and Built Environment, Deakin University, Victoria, Australia; Queens College CUNY

## Abstract

We characterized the complete genome of a lytic Dickeya chrysanthemi bacteriophage, DchS19, which was isolated from a soil sample in Sungai Petani, Kedah, Malaysia. The phage, from the *Autographviridae* family, has a 39,149-bp double-stranded DNA genome containing 49 protein-coding genes and shares 94.65% average nucleotide identity with *Erwinia* phage pEp_SNUABM_12.

## ANNOUNCEMENT

Soft rot disease, which is caused by macergens such as Dickeya chrysanthemi, is a major problem in agriculture, resulting in 15 to 30% of crop losses annually (1, 2). The current control measures include metaphylactic application of antibiotics and pesticides that are nonspecific against the pathogen, which results in disruption of natural commensals in the environment (3). Through our study, we proved that phage therapy can be applied as a targeted alternative (4). However, it is important to study the genome of the phage used for therapy to ensure that it does not carry any deleterious genes such as genes related to lysogeny and pathogenicity (5). In line with this, we successfully isolated and sequenced a *Dickeya* phage, DchS19, from a soil sample obtained in the vicinity of soft rot-infected plants in Kedah, Malaysia (5°41′34.1″N, 100°29′36.1″E). The complete genome sequence of the phage is reported here.

**FIG 1:**
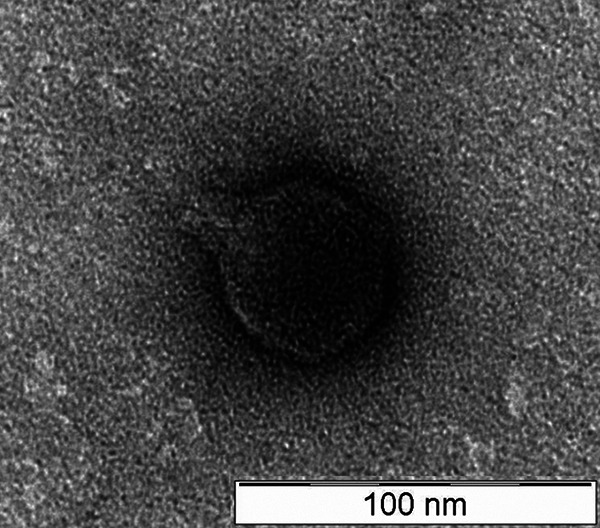
Transmission electron microscopic image of phage DchS19.

Phage DchS19 was isolated using the enrichment method with Dickeya chrysanthemi ATCC 11663 as its host (6). Subsequently, the phage was enriched to a high titer using the double-overlay agar technique (7). Phage morphology was then visualized using 1% (wt/vol) uranyl acetate and observed under a transmission electron microscope at 40 kV (Fig. 1). The phage possesses an isometric head (diameter, 49.33 ± 2.3 nm) and a cone-shaped tail stub (length, 20 nm).

Phage DchS19 DNA was extracted using the phenol-chloroform method (8) and quantified with a Qubit fluorometer. The total DNA was subjected to processing with a Nextera DNA Flex library preparation kit and sequenced using the Illumina MiSeq platform, which yielded 251,912 reads with 300-bp paired-end sequences. Raw reads were assessed using FastQC v0.11.9 (9) and then trimmed using Trimmomatic v0.39 with the following parameters: SLIDINGWINDOW:4:20, HEADCROP:10, CROP:30 (10). Following this, a total of 100,000 quality-controlled reads were subsampled using seqtk (11), and the genome was assembled using Unicycler v0.4.8 with default settings (12). The length of the assembled genome was 39,149 bp, with a GC content of 51.34%. Assembly evaluation using Bowtie2 v2.4.4 (13) revealed that a total of 98.10% reads mapped back to the genome, with an average coverage of 111×. PhageLeads (14) analysis showed that DchS19 is a lytic phage, with no lysogenic factors or antibiotic resistance genes in the genome.

The annotation of the assembled genome was performed using Prokka v1.12 (15), which predicted the presence of 49 protein-coding genes; 22 had putative functions and 27 were hypothetical proteins, with no tRNAs. Further analysis indicated that the phage had <95% average nucleotide identity (ANI) with all other phages reported in the NCBI nucleotide database, suggesting that the phage is a new species (16). In accordance with the International Committee on Taxonomy of Viruses (ICTV) and genome-based classification, the phage belongs to the genus *Ningirsuvirus*, in the order *Caudovirales* and the family *Autographviridae*.

### Data availability.

The complete genome of phage DchS19 has been deposited in the GenBank database under the accession number ON287378. The associated BioProject, SRA, and BioSample accession number are PRJNA809096, SRR18094404, and SAMN26142374, respectively.

## References

[1] Bhat KA, Masood SD, Bhat NA, Bhat MA, Razvi SM, Mir MR, Akhtar S, Wani N, Habib M. 2010. Current status of post harvest soft rot in vegetables: a review. Asian J Plant Sci 9:200–208. doi:10.3923/ajps.2010.200.208.

[2] Rizzo DM, Lichtveld M, Mazet JAK, Togami E, Miller SA. 2021. Plant health and its effects on food safety and security in a One Health framework: four case studies. One Health Outlook 3:6. doi:10.1186/s42522-021-00038-7. 33829143 PMC8011176

[3] Aktar M, Sengupta D, Chowdhury A. 2009. Impact of pesticides use in agriculture: their benefits and hazards. Interdiscip Toxicol 2:1–12. doi:10.2478/v10102-009-0001-7. 21217838 PMC2984095

[4] Khor JR, Jothi SJ, Kurunathan S, Lee KY, Lee SY, Chan S-Y. 2021. Biocontrol capability of bacteriophages against soft rot disease caused by *Dickeya chrysanthemi*. Malays J Microbiol 17:615–623. doi:10.21161/mjm.211140.

[5] Doub JB. 2021. Risk of bacteriophage therapeutics to transfer genetic material and contain contaminants beyond endotoxins with clinically relevant mitigation strategies. Infect Drug Resist 14:5629–5637. doi:10.2147/IDR.S341265. 34992389 PMC8711558

[6] Cross T, Schoff C, Chudoff D, Graves L, Broomell H, Terry K, Farina J, Correa A, Shade D, Dunbar D. 2015. An optimized enrichment technique for the isolation of *Arthrobacter* bacteriophage species from soil sample isolates. J Vis Exp 52781. doi:10.3791/52781. PMC4541497 25938576

[7] Jakočiūnė D, Moodley A. 2018. A rapid bacteriophage DNA extraction method. Methods Protoc 1:27. doi:10.3390/mps1030027. PMC6481073 31164569

[8] Hyman P, Abedon ST. 2009. Practical methods for determining phage growth parameters. Methods Mol Biol 501:175–202. doi:10.1007/978-1-60327-164-6_18. 19066822

[9] Trivedi UH, Cézard T, Bridgett S, Montazam A, Nichols J, Blaxter M, Gharbi K. 2014. Quality control of next-generation sequencing data without a reference. Front Genet 5:111. doi:10.3389/fgene.2014.00111. 24834071 PMC4018527

[10] Bolger AM, Lohse M, Usadel B. 2014. Trimmomatic: a flexible trimmer for Illumina sequence data. Bioinformatics 30:2114–2120. doi:10.1093/bioinformatics/btu170. 24695404 PMC4103590

[11] Li H. 2022. seqtk: toolkit for processing sequences in FASTA/Q formats. https://github.com/lh3/seqtk.

[12] Wick RR, Judd LM, Gorrie CL, Holt KE. 2017. Unicycler: resolving bacterial genome assemblies from short and long sequencing reads. PLoS Comput Biol 13:e1005595. doi:10.1371/journal.pcbi.1005595. 28594827 PMC5481147

[13] Langmead B, Salzberg SL. 2012. Fast gapped-read alignment with Bowtie 2. Nat Methods 9:357–359. doi:10.1038/nmeth.1923. 22388286 PMC3322381

[14] Yukgehnaish K, Rajandas H, Parimannan S, Manickam R, Marimuthu K, Petersen B, Clokie MRJ, Millard A, Sicheritz-Pontén T. 2022. PhageLeads: rapid assessment of phage therapeutic suitability using an ensemble machine learning approach. Viruses 14:342. doi:10.3390/v14020342. 35215934 PMC8879740

[15] Seemann T. 2014. Prokka: rapid prokaryotic genome annotation. Bioinformatics 30:2068–2069. doi:10.1093/bioinformatics/btu153. 24642063

[16] Turner D, Kropinski AM, Adriaenssens EM. 2021. A roadmap for genome-based phage taxonomy. Viruses 13:506. doi:10.3390/v13030506. 33803862 PMC8003253

